# Attention-Deficit/Hyperactivity Disorder Diagnosis, Treatment, and Telehealth Use in Adults — National Center for Health Statistics Rapid Surveys System, United States, October–November 2023

**DOI:** 10.15585/mmwr.mm7340a1

**Published:** 2024-10-10

**Authors:** Brooke S. Staley, Lara R. Robinson, Angelika H. Claussen, Samuel M. Katz, Melissa L. Danielson, April D. Summers, Sherry L. Farr, Stephen J. Blumberg, Sarah C. Tinker

**Affiliations:** ^1^National Center on Birth Defects and Developmental Disabilities, CDC; ^2^Epidemic Intelligence Service, CDC; ^3^Oak Ridge Institute for Science and Education, Oak Ridge, Tennessee; ^4^National Center for Health Statistics, CDC.

**Summary**

**What is already known about this topic?**

Attention-deficit/hyperactivity disorder (ADHD) is a common childhood disorder that can continue into adulthood, but there is limited information about diagnosis and treatment in adults.

**What is added by this report?**

In 2023, an estimated 15.5 million U.S. adults had an ADHD diagnosis, approximately one half of whom received their diagnosis in adulthood. Approximately one third of adults with ADHD take stimulant medication; 71.5% had difficulty filling their prescription because the medication was unavailable. Approximately one half of adults with ADHD have ever used telehealth for ADHD services.

**What are the implications for public health practice?**

ADHD affects many adults. Information on diagnosis and treatment helps the development of clinical care guidelines and regulatory decision-making around medication shortages and telehealth for ADHD.

## Abstract

Attention-deficit/hyperactivity disorder (ADHD) is a neurodevelopmental disorder that develops during childhood and can last into adulthood. Data from the National Center for Health Statistics Rapid Surveys System collected during October–November 2023 were used to estimate the prevalence of ADHD diagnosis and treatment among U.S. adults. In 2023, an estimated 15.5 million U.S. adults (6.0%) had a current ADHD diagnosis based on self-report; approximately one half received the diagnosis at age ≥18 years. Approximately one third of adults with ADHD took a stimulant medication to treat their ADHD in the previous year, 71.5% of whom had difficulty getting their ADHD prescription filled because it was unavailable. Approximately one half of adults with ADHD have ever used telehealth for ADHD-related services. Telehealth might have benefits for persons with ADHD, including helping them access behavioral treatment or medication prescriptions for ADHD. This report provides national estimates of the prevalence and treatment of ADHD among U.S. adults to help guide clinical care and regulatory decision-making for ADHD among U.S. adults.

## Introduction

Worldwide, approximately 2%–5% of adults experience attention-deficit/hyperactivity disorder (ADHD) symptoms such as inattention, hyperactivity, and impulsivity (*1*,*2*). However, recent data on ADHD diagnosis and treatment among adults in the United States are limited; no national data exist on ADHD treatment in U.S. adults, and national prevalence estimates of current ADHD in adults rely on data from 2003.^†^ Telehealth policies implemented during the COVID-19 pandemic expanded access to ADHD diagnosis and treatment, including medication (*3*). Pharmacotherapy is a first-line treatment for adults with ADHD (*2*), and prescribing of stimulant medication has increased since the COVID-19 pandemic began (*4*). Shortages of stimulant medications^§^ in the United States have affected many persons with ADHD who rely on pharmacotherapy to appropriately treat their ADHD (*2*,*5*). Timely data are needed to develop clinical guidelines, and guide decision-making for policies, including regulation concerning stimulant prescription and telehealth access for ADHD in U.S. adults.

## Methods

### Rapid Survey System: Survey Panels and Sample

The National Center for Health Statistics (NCHS) Rapid Surveys System (RSS) approximates national representation of the U.S. adult population based on self-reported health data from two commercial online survey panels: NORC at the University of Chicago’s AmeriSpeak Panel (*1*) and Ipsos’s KnowledgePanel (*2*). These cross-sectional samples are surveyed simultaneously using the same RSS questionnaire, conducted online and by telephone, and are then combined (*6*). To reduce coverage and nonresponse biases, responses are weighted and calibrated to the estimates from the second quarter of the 2023 National Health Interview Survey to reflect the total population of U.S. adults aged ≥18 years. The RSS Round 2 (RSS-2), fielded during October–November 2023, includes data from 7,046 completed interviews (*6*). More details on RSS and incorporated panels are available at https://www.cdc.gov/nchs/rss/rss-topics.html; the RSS-2 brief technical note is available at https://www.cdc.gov/nchs/data/rss/round2/technical-notes.pdf. The cumulative response rates of the two commercial panels were 3.8% and 4.0%, and the RSS-2 overall completion rate was 37.2% (*6*).

### RSS-2 ADHD

Adults with current ADHD were identified using two survey questions: “Have you ever been diagnosed with attention-deficit/hyperactivity disorder, or ADHD, by a doctor or other health professional?” and, if so, “Do you currently have ADHD?” (https://www.cdc.gov/nchs/data/rss/round2/questionnaire.pdf). Adults reporting current ADHD received follow-up questions regarding receipt and type of treatment, type of medication use (categorized as stimulant or nonstimulant medications), difficulty obtaining prescription medication, and use of telehealth services for their ADHD care. Demographic variables included age, age at diagnosis (<18 years versus ≥18 years), gender, education, race and ethnicity, household income as a percentage of the federal poverty level, insurance status, and metropolitan status; these data were collected before the survey through panel-specific profile assessments that are harmonized for inclusion in RSS-2 data (*6*). SAS-callable SUDAAN (version 11.0.3; RTI International) was used to conduct all analyses. Variances were computed using the Taylor linearization method. This activity was reviewed by CDC, deemed not research, and was conducted consistent with applicable federal law and CDC policy.^¶^

## Results

### Prevalence of ADHD Among U.S. Adults

An estimated 6.0% of adults had a current ADHD diagnosis, equivalent to one in 16, or approximately 15.5 million U.S. adults (Table 1). When compared with adults who have never received a diagnosis of ADHD, those with current ADHD were more likely to be aged <50 years (84.5% versus 51.2%), less likely to have a bachelor’s degree or higher (28.1% versus 35.3%), less likely to be non-Hispanic Black or African American (7.4% versus 12.9%), more likely to be non-Hispanic White (70.4% versus 61.4%), and more likely to have a household income below the federal poverty level (22.1% versus 12.3%).

**TABLE 1:** Demographic distribution among adults with current attention-deficit/hyperactivity disorder and adults who have never received an attention-deficit/hyperactivity disorder diagnosis — National Center for Health Statistics Rapid Surveys System, United States, October–November 2023

Characteristic	With current ADHD diagnosis*		Never received an ADHD diagnosis*	
	Unweighted no.	Weighted^†^ % (95% CI)	Unweighted no.	Weighted^†^ % (95% CI)
**Total^§^**	**444**	**6.0 (5.3–6.8)**	**6,441**	**92.2 (91.4–93.0)**
**Age group at time of survey,^¶^ yrs**				
18–24	70	21.7 (16.4–27.8)	363	10.1 (8.9–11.3)
25–49	278	62.8 (56.6–68.6)	2,338	41.1 (39.6–42.6)
50–64	69	10.6 (7.9–14.0)	1,790	25.0 (23.7–26.3)
≥65	27	4.9 (2.7–8.1)	1,950	23.9 (22.8–25.0)
**Gender^¶^**				
Female	214	44.2 (38.0–50.5)	3,502	51.7 (50.1–53.2)
Male	230	55.8 (49.5–62.0)	2,939	48.3 (46.8–49.9)
**Education^¶^**				
High school graduate or less	146	41.8 (35.6–48.3)	1,816	37.5 (36.0–39.0)
Some college	154	30.1 (24.6–36.0)	2,046	27.2 (25.9–28.4)
Bachelor's degree or above	144	28.1 (23.1–33.5)	2,579	35.3 (34.0–36.7)
Race and ethnicity^¶,^**				
Black or African American	30	7.4 (4.5–11.4)	673	12.9 (11.8–14.1)
White	301	70.4 (64.2–76.0)	4,308	61.4 (59.8–63.0)
Hispanic or Latino	75	16.6 (12.0–22.1)	865	17.5 (16.2–18.8)
Other	34	5.6 (3.3–8.8)	544	8.1 (7.3–9.0)
**Household income as a percentage of FPL^¶^**				
<100	99	22.1 (17.3–27.6)	710	12.3 (11.2–13.4)
100 to <200	74	14.5 (10.5–19.2)	1,117	17.6 (16.4–18.8)
200 to <400	114	23.3 (18.3–29.0)	1,795	26.6 (25.3–27.9)
≥400	157	40.0 (34.0–46.4)	2,819	43.6 (42.1–45.1)
**Metropolitan status^¶^**				
Metro area	372	83.0 (77.9–87.3)	5,529	86.3 (85.2–87.3)
Nonmetro area	72	17.0 (12.7–22.1)	912	13.7 (12.7–14.8)

**Abbreviations:** ADHD = attention-deficit/hyperactivity disorder; FPL = federal poverty level.

* Respondents were asked, “Have you ever been diagnosed with attention-deficit/hyperactivity disorder, or ADHD, by a doctor or other health professional?” Those who responded “yes” were then asked, “Do you currently have ADHD?”

^†^ Weighted to reflect the total population of U.S. adults aged ≥18 years, based on estimates from the second quarter of the 2023 National Health Interview Survey.

**^§^** Adults who reported previous but not current ADHD diagnosis (129; 1.8%) are not included in the table. Adults who did not answer the initial diagnosis question (32) were excluded from the analysis. Row percentage is reported in the table for this variable.

**^¶^** This information was not collected as part of the Rapid Surveys System survey but came from the panel’s profile data. Column percentages are reported in the table for these variables.

** Persons of Hispanic or Latino (Hispanic) origin might be of any race but are categorized as Hispanic; all racial groups are non-Hispanic. Only the Other group included multiple races; the other groups consisted of a single racial group. Data on race and ethnicity were missing for four respondents with a current ADHD diagnosis, and 51 respondents who have never received an ADHD diagnosis.

### ADHD Diagnosis and Treatment

More than one half of adults with ADHD (55.9%) received their diagnosis during adulthood (age ≥18 years) (Table 2). At the time of the survey, approximately one third of adults with ADHD were not receiving any treatment (36.5%), while another one third were receiving both medication and counseling or behavioral treatment (35.2%). Approximately one half of adults (50.4%) with ADHD were prescribed medication to treat their ADHD during the previous 12 months. Approximately one third of adults with current ADHD reported taking prescription stimulant ADHD medication during the previous 12 months (33.4%); nonstimulant ADHD medication use was less common (5.9%). Among adults who reported taking a stimulant medication, 71.5% reported difficulty getting their ADHD prescription filled during the previous 12 months because their medication was not available.

**TABLE 2:** Age at diagnosis and treatment among 444 adults with current attention-deficit/hyperactivity disorder — National Center for Health Statistics Rapid Surveys System, United States, October–November 2023

Characteristic	Unweighted no.	Weighted* % (95% CI)
**Age group at diagnosis,^†^ yrs**		
<18	164	44.1 (37.7–50.6)
≥18	275	55.9 (49.4–62.3)
**ADHD treatment during the previous 12 mos^§^**		
None	152	36.5 (30.5–42.8)
Medication and counseling or behavioral treatment	157	35.2 (29.2–41.5)
Counseling or behavioral treatment only	59	13.3 (9.4–18)
Medication only	75	15.1 (11.3–19.6)
**Was prescribed medication to treat ADHD during the previous 12 mos^¶^**		
Yes	232	50.4 (43.9–56.9)
No	210	49.6 (43.1–56.1)
Reported taking a prescribed stimulant ADHD medication during the previous 12 mos**		
Yes	152	33.4 (27.5–39.7)
No	292	66.6 (60.3–72.5)
Reported taking a prescribed nonstimulant ADHD medication during the previous 12 mos**		
Yes	27	5.9 (3.4–9.4)
No	417	94.1 (90.6–96.6)
**Reported having difficulty getting ADHD prescription filled during the previous 12 mos because their ADHD medication was not available**		
Among all adults who reported taking any ADHD medication^††^	141	61.8 (52.9–70.1)
Among adults who reported taking stimulant ADHD medication^§§^	108	71.5 (60.9–80.6)

**Abbreviation:** ADHD = attention-deficit/hyperactivity disorder.

* Weighted to reflect the total population of U.S. adults aged ≥18 years, based on estimates from the second quarter of the 2023 National Health Interview Survey.

^†^ Respondents who self-reported ever being diagnosed with ADHD by a doctor or health professional were asked, “How old were you when a doctor or other health professional first diagnosed you with ADHD?” Data for age at diagnosis were missing for five adults with current ADHD.

^§^ Respondents who self-reported having ADHD currently were asked, “During the past 12 months, did you receive counseling or therapy from a mental health professional to help you with your ADHD?” and “During the past 12 months, were you prescribed medication to help you with your ADHD?” Data for ADHD treatment were missing for one adult with current ADHD.

^¶^ Respondents who reported having ADHD currently were asked, “During the past 12 months, were you prescribed medication to help you with your ADHD?” Data on whether a medication to treat ADHD was prescribed during the previous 12 months were missing for two respondents.

** Respondents who reported having ADHD currently were asked, “During the past 12 months, what prescription medications did you take to help you with ADHD? Please do not list any medications you were prescribed but did not take.”

^††^ Sample restricted to the 232 adults who reported that they were prescribed medication to treat their ADHD during the previous 12 months.

^§§^ Sample restricted to the 152 adults who reported taking a stimulant medication to treat their ADHD during the previous 12 months.

### Telehealth Use for ADHD

Almost one half of adults with ADHD (46.0%) reported ever receiving telehealth services for their condition (Table 3). Approximately one in 11 adults (8.9%) received their diagnosis via telehealth only, and an additional one in 10 (9.5%) received their diagnosis through a combination of in-person and telehealth visits. Since the start of the COVID-19 pandemic (i.e., March 2020), approximately one third of adults with current ADHD used telehealth to obtain a prescription for ADHD medication (30.5%) or to receive counseling or therapy for ADHD (30.8%).

**TABLE 3:** Telehealth use among 444 adults with current attention-deficit/hyperactivity disorder — National Center for Health Statistics Rapid Surveys System, United States, October–November 2023

Characteristic	Unweighted no.	Weighted* % (95% CI)
**Ever received telehealth services for ADHD^†^**		
Yes	201	46.0 (39.9–52.3)
No	240	54.0 (47.7–60.1)
**ADHD diagnosis receipt^§^**		
Only telehealth visits	46	8.9 (6.0–12.5)
A mix of in-person and telehealth visits	36	9.5 (6.2–13.7)
Only in-person visits	357	81.7 (76.6–86.0)
**At any time since the start of the COVID-19 pandemic (i.e., March 2020) used any telehealth visit** ^¶^		
With a doctor, nurse, or other health professional to get a prescription for medication to help their ADHD	136	30.5 (24.9–36.6)
To receive counseling or therapy to help with their ADHD	141	30.8 (25.1–37.0)

**Abbreviation:** ADHD = attention-deficit/hyperactivity disorder.

* Weighted to reflect the total population of U.S. adults aged ≥18 years, based on estimates from the second quarter of the 2023 National Health Interview Survey.

^†^ Respondents who self-reported having ADHD currently were asked, “Have you ever received any telehealth services for ADHD? That is, have you ever talked about your ADHD with a doctor, nurse, or other health professional by video or by phone?” Data for ever using telehealth services for ADHD were missing for three adults who had a current ADHD diagnosis.

^§^ Respondents who self-reported having used telehealth to receive ADHD care were asked, “Were you diagnosed with ADHD during telehealth visits, in-person visits, or a combination of both?” Missing data for two respondents and denominator was restricted to adults who reported ever using telehealth services for ADHD.

^¶^ Respondents could select both items; therefore, percentages are not exclusive.

## Discussion

This analysis of a nationally representative sample of U.S. adults found that in 2023, an estimated 15.5 million (6.0%) had a current ADHD diagnosis, approximately one half of whom received their diagnosis during adulthood. Results highlight the magnitude of ADHD as a public health issue across the life course. Approximately one third of adults with current ADHD are not receiving any ADHD treatment. Among those receiving stimulant pharmacotherapy, seven in 10 reported difficulty obtaining their ADHD medication because it was not available. Approximately one half of adults with current ADHD have ever used telehealth for ADHD services.

Diagnostic criteria for ADHD require evidence of symptoms before age 12 years (*7*), but actual diagnosis might occur years beyond symptom onset. These data suggest diagnosis in adulthood is common. Although the majority of adults with current ADHD received counseling or medication treatment for their ADHD in the previous year, approximately one third did not receive any type of treatment. ADHD pharmacotherapy is associated with reduced social and emotional impairment, unintentional injuries, substance use disorders, and risk of death due to unnatural causes (*2*,*5*).

The finding that 71.5% of adults who reported taking a stimulant medication had difficulty getting their ADHD prescription filled during the previous 12 months highlights the importance of ensuring an adequate supply of these medications. A 2024 CDC Health Advisory** conveyed that medication shortages and major disruptions to ADHD provider access increase concerns about risk for injury and overdose. Patients experiencing these difficulties might seek medication outside the regulated health care system, increasing their risk for overdose because of the prevalence of counterfeit pills in the illegal drug market, which might contain unexpected substances such as fentanyl.

The availability of clinical care guidelines for adults with ADHD could improve standards of care and associated health outcomes for this population (*8*). Reducing delays in diagnosis and treatment access could improve ADHD symptoms and long-term health risks for adults with the condition (*2*,*3*).

Research using health care claims data suggests that approximately one half of adults with ADHD received their ADHD care via telehealth, and that adults with ADHD use telehealth approximately twice as frequently as do those without ADHD (*9*). Similarly, the current data indicate that approximately one half of adults with ADHD have ever used telehealth for ADHD care. In March 2023, the Drug Enforcement Administration and the U.S. Department of Health and Human Services extended COVID-19 flexibilities regarding stimulant prescribing via telehealth^††^ without an initial in-person medical evaluation through December 31, 2024. Findings in this report provide information on the size of the affected population for potential rule changes, and if the exception is not extended, provide information that can help providers prepare for increased in-person health care demands.

Telepsychiatry guidelines for ADHD care acknowledge the potential benefits and risks associated with use of telehealth for ADHD care. Benefits include reduced time and effort, especially given the organizational challenges faced by persons with ADHD; increased access, especially in some geographic areas; and reduced wait times. Risks include concerns about the quality of care, such as accuracy of diagnosis and potential for misuse or diversion of prescription medication, and lack of access to technology by some populations. Experts on ADHD treatment suggest that the benefits of increased access to diagnosis and treatment via telehealth outweigh the risks of undiagnosed and untreated ADHD (*3*). Evaluating, monitoring, and identifying standards for quality telehealth implementation have been demonstrated to help maximize these benefits and limit risks (*10*).

### Limitations

The findings in this report are subject to at least three limitations. First, self-reports of ADHD diagnosis might be subject to recall and reporting biases and were not validated against medical records. Second, surveys with commercial online panels have low response rates and might underrepresent certain subpopulations, increasing the potential for nonresponse bias. Nonresponse bias in RSS is reduced through innovative weighting approaches and calibration of the data to benchmark NCHS surveys, with comparisons to the National Health Interview Survey suggesting low bias for prevalence estimates of chronic health conditions (*6*). The data are cross-sectional and cannot be used to examine trends over time. Finally, the sociodemographic and geographic data were collected before the RSS survey administration, which could have affected the demographic distribution for some variables such as age, education, household income, and metropolitan status (*6*).

### Implications for Public Health Practice

Public health professionals can use the findings from this report to better understand the prevalence of ADHD in adulthood, how adults obtain ADHD care, the potential gaps or delays in diagnosis, and the magnitude of treatment needs. As policies are currently developed and evaluated related to ADHD clinical care for adults, access to prescription stimulant medications, and flexibilities related to telehealth, these results can guide clinical care and regulatory decision-making.

## Footnotes

†
https://www.nimh.nih.gov/health/statistics/attention-deficit-hyperactivity-disorder-adhd

§
https://www.fda.gov/drugs/drug-safety-and-availability/fda-announces-shortage-adderall

¶ 45 C.F.R. part 46, 21 C.F.R. part 56; 42 U.S.C. Sect. 241(d); 5 U.S.C. Sect. 552a; 44 U.S.C. Sect. 3501 et seq.

**
https://emergency.cdc.gov/han/2024/pdf/CDC_HAN_510.pdf

††
https://www.federalregister.gov/documents/2023/10/10/2023-22406/second-temporary-extension-of-covid-19-telemedicine-flexibilities-for-prescription-of-controlled
